# Modelling of heat addition to near-critical and supercritical fluids

**DOI:** 10.1038/s41598-025-32459-z

**Published:** 2025-12-26

**Authors:** Grazia Lamanna, Christoph Steinhausen

**Affiliations:** Institute of Aerospace Thermodynamics, University of Stuttgart, Stuttgart, 70569 Germany

**Keywords:** Engineering, Mathematics and computing, Physics

## Abstract

This work presents a coupled flow model to describe heat addition to near-critical and supercritical fluids. In a first approximation only forced convection is considered, while buoyancy forces, viscous and volume expansion losses are neglected. The steady, one-dimensional balance equations are solved simultaneously through an iterative procedure, taking into account real gas effects. The analysis shows that the intrinsic high compressibility of near-critical fluids strongly reduces the region of validity for the incompressible flow assumption. Indeed, depending on the initial conditions, compressible flow effects may occur at Mach numbers below 0.1. For a given mass flux, the occurrence of heat transfer deterioration (HTD) correlates directly with the maximum amount of heat ($Q_{max}$) that a compressible flow can absorb at a specific local Mach number. For near-critical fluids, $Q_{max}$ depends upon two parameters, namely the heat flux to mass flux ratio and the thermal dilatation. The latter induces not only the early inception of compressibility effects, but it also poses an additional volumetric constraint to $Q_{max}$. Indeed, in confined flows the enhancement of the thermal dilatation parameter strongly limits the capability of the flow to increase the local mass flux by expanding the flow, which eventually decreases the $Q_{max}$ value. These findings provide a generalization of the well-known dependence of $Q_{max}$ upon the local Mach number for an ideal gas. The local enhancement of the thermal dilatation also explains the counterintuitive cooling of the fluid upon heat addition. Overall, our analysis advocates for a more comprehensive flow analysis in the design of regenerative cooling systems.

## Introduction

**Fig. 1:**
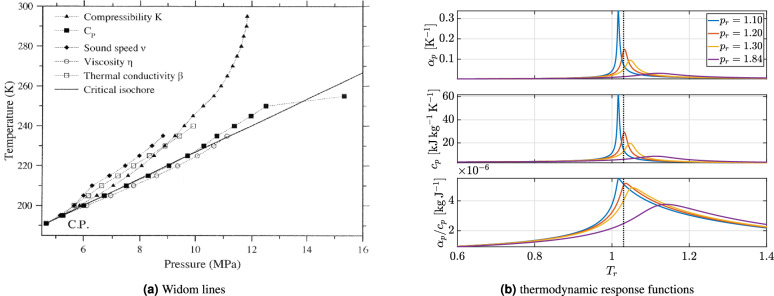
Methane (CH$_4$) behavior in the supercritical region: (**a**) overview of Widom lines. From The Liquid and Supercritical Fluid States of Matter by John E. Proctor, Copyright ©  (2022) by CRC Press. Reproduced by permission of Taylor & Francis Group. Here $C_p$ denotes the isobaric molar heat capacity. (**b**) Variations of the thermodynamic response functions $\alpha _p$ and $c_p$ for an isobaric temperature increase.

The supercritical fluid (SCF) is commonly considered as a unique state with intermediate properties of the liquid, gas and solid phases^1^. This view is supported by the fact that, on the continuum scale, all thermodynamical measurable quantities exhibit a continuous evolution along any pressure/temperature (*P*-*T*) path in the supercritical region. A notable characteristic of the supercritical region is the existence of narrow transitions, where both thermodynamic properties and the dynamic behavior of SCFs change rapidly in a narrow range of pressures and/or temperatures. Examples of such transitions are the Joule-Thomson, Boyle or Amagat inversion curves. Their derivations from first principles and physical meaning can be found in Proctor^1^. Additional transition curves include the Frenkel and Widom lines. Each Widom line denotes the locus of maxima (or minima) of different thermodynamic parameters. Examples are the isobaric heat capacity $c_p = T (\partial s /\partial T)_p$ and the isobaric thermal expansion coefficient $\alpha _p = 1/v \,(\partial v /\partial T)_p$, where *s* is the specific entropy, *T* the temperature, *p* the pressure and *υ* the specific volume. As shown in Fig. 1a, the Widom lines emanate all from the critical point as a prolongation of the vapor-liquid equilibrium curve and rapidly diverge as the pressure and temperature increase^1^. Currently, both the Frenkel and Widom lines are at the center of a controversial debate on the occurrence of positive sound dispersion (PSD), which marks a crossover from the liquid-like to the gas-like relaxation dynamics in response to small (acoustic) disturbances. A recent, comprehensive review on the different types of relaxation processes occurring in the supercritical region can be found in Lamanna^2^.

This brief overview shows that the behavior of SCFs is until today poorly understood. Specifically, it is still unclear how the transition from the liquid to the supercritical state occurs, namely which properties of the liquid state persist in the supercritical region. Second, close to the critical point, it is not clear how to model the SCF’s response to perturbations in pressure and/or temperature due the simultaneous and rapid variation of several thermodynamic response function across the Widom lines, as shown in Fig. 1b. A practical example of such difficulty is the so-called heat transfer deterioration (HTD). HTD denotes a diminished capacity of SCFs to absorb heat and it is typically encountered in regenerative cooling applications for liquid rocket engines^3,4^, in natural circulation loops for nuclear reactors and solar thermal systems^5^ and in transcritical CO$_2$ Rankine cycles for waste heat recovery^6^. All these technical applications differ in the type of convective transport, i.e. forced or natural convection, and the associated inlet mass flux *G*. The relevance of this parameter on the fluid capacity to absorb heat is discussed later in the “Result” section. At this stage, it is important to point out that there is currently no unique definition for marking the onset of HTD. Hereafter, the most common criteria are reported. In all the above mentioned systems, a rapid local increase in wall temperature is experimentally detected. This indicates that the heat transfer performances are impaired and marks the onset of HTD. An alternative definition is based on the monitoring of the heat transfer coefficient. The sudden increase in wall temperature corresponds to a decrease along the axial direction of the heat transfer coefficient, thus marking the onset of HTD^4,7^. Experimental studies have been conducted in horizontal, vertical and helically coiled pipes, having as test fluids supercritical water^8,9^, carbon dioxide (sCO$_2$)^10^, methane (sCH$_4$)^3,4,7^ and organic fluids^11^. These studies demonstrate that HTD is not unique to a specific class of fluids and correlates directly with the rapid variations of the response functions $c_p, \alpha _p$ in the Widom region. Longmire & Banuti^12^ proposed a direct correlation between HTD and the pseudo-boiling transition, which is based on an analogy with subcritical nucleate boiling along the $c_p$-Widom line. Their model foresees heat transfer enhancement at the pseudo-boiling temperature $(T_{pb})$ due to the associated peak in thermal conductivity and specific isobaric heat capacity. Upon a further increase in the fluid temperature, HTD rapidly sets in owing to the formation of an insulating gaseous layer along the heated wall with decreased values in density, $c_p$ and thermal conductivity. Experimental studies indirectly corroborated these theoretical considerations by showing that, in heated channels, HTD typically occurs when the pseudo-boiling temperature ($T_{pb}$), evaluated at the $c_p$-Widom line, is intermediate between the bulk temperature $T_b$ and the wall temperature $T_w$ (alias $T_b< T_{pb} < T_w$)^6,10,11^. In a recent paper, Nasuti and Pizzarelli^4^ pointed out that the previous requirement is a necessary but not sufficient condition, since the onset of HTD largely depends upon the applied heat flux to mass flux ratio ($\dot{q}_w / G$) and pressure. Indeed, mitigation measures include the increase of the mass flux or the generation of additional turbulence through wall roughness or by installations of ribs/grooves to promote turbulent mixing. In order to explain this interdependency, an extended model for the shear stress was proposed that includes both the effects of the variable thermophysical properties and of the ratio $\dot{q}_w / G$. Specifically, a non-dimensional group was introduced $\dot{q}_w/(G f_w) (\alpha _p /c_p)_b$ with $f_w$ the friction factor and the subscript *b* indicating that all properties are evaluated at the bulk state. When the non-dimensional group exceeds a threshold value, the onset of HTD is predicted. Physically, the overall goal is to model the decrease in shear stress close to the wall, thereby mimicking the depletion of turbulent kinetic energy and the subsequent increase of the turbulent thermal resistance to heat transfer, i.e. HTD. The above-mentioned non-dimensional group shows that HTD can be prevented either by increasing the mass flux *G* or by increasing the bulk temperature above $T_{pb}$, leading to a reduction of $(\alpha _p /c_p)_b$. The effects of the mass flux were further discussed in the review of Xie et al.^13^. The authors investigated a wide range of inlet conditions in the range of $10^2< G < 10^4$ (kg m$^{-2}$ s$^{-1}$) and corroborated the findings of Pizzarelli and collaborators^4,7,14^. Namely, large mass fluxes (i.e. $G > 10^3$ kg m$^{-2}$ s$^{-1}$) weaken the unconventional heat transfer behavior and the effect of buoyancy forces can be ignored. Additionally, Ren et al.^15^ proposed pulsating flow as a method to mitigate HTD in laminar pipe flows.

An interesting additional feature is observed in technical application where buoyancy is no longer negligible, such as natural circulation (NC) loops or transcritical CO$_2$ Rankine cycles. In these applications, upon the onset of HTD a further increase in the heat flux may lead to the onset of an oscillatory, periodic flow^5,6,8,9^. Different modeling approaches have been proposed to explain this phenomenon. Zhang et al.^6^ proposed a shear stress reconstruction model that takes into account the competitive effects of thermal acceleration due to heat addition and buoyancy. Specifically, under heating conditions, the density gradient in radial direction can produce a strong buoyancy effect in the presence of gravity and promote the production of turbulent kinetic energy. This corresponds to a regime of partial re-laminarization and mitigates HTD. Simultaneously, thermal acceleration is caused by the increase in flow velocity associated to the rapid volume expansion. This leads to a full re-laminarization of the flow near the wall, thereby weakening the turbulent kinetic energy intensity and repristinating HTD. Their interplay induces a periodic transition between partial re-laminarization and full re-laminarization, respectively, and results in a periodic oscillation of the local wall temperature, outlet temperature, pressure, and mass flux. Above a certain heat flux threshold, the steady state is recovered again due to a full re-laminarization of the flow. For natural circulation loops, a different modeling approach was proposed by Debrah et al.^9^ based on the theoretical considerations of Ambrosini and Sharabi^8^. The authors solved the one-dimensional, (un)steady balance equations for mass, momentum and internal energy with the inclusion of buoyancy and friction forces. In the steady-state solution, the mass flow rate is the result of the assigned heating level and of the balance between friction and buoyancy forces. In the simulations, an additional constraint is needed to satisfy the overall energy balance of the loop, which implies equality of inlet (heater) and outlet (cooler) energy supply. The loop energy balance is no longer valid upon the onset of oscillatory flow. The latter can be stabilized by increasing the coolant flow rate, as shown in the 1D simulations of Sharma et al.^16^. In this context, it is important to point out that, in actual NC loops, the heat flux in the cooler is applied indirectly by imposing the coolant flow rate. In the experiments^16^, the instability was observed for a very narrow window of applied powers in combination with low coolant flow rates at operating temperatures close to the pseudo-boiling temperature, where the isobaric thermal expansion coefficient of the fluid is the highest. Moreover, the agreement between experiments and simulation in predicting the onset of the instability could be substantially improved by taking into account the pipe wall thermal capacitance^16^.

The preceding discussion has revealed the following important aspects. Irrespective of the driving force (buoyancy or pressure gradient), the onset of HTD and of the oscillatory flow in NC loops is strongly related to the heat flux to mass flux ratio (alias the NCL power-to-flow ratio) and the ratio ($\alpha _p / c_p$). Second, despite the strong simplifications, one-dimensional modeling approaches that solve simultaneously mass, momentum and energy balance are capable of reproducing the experimentally observed instabilities. These findings closely correlate with a well-known theory for modeling heat addition to compressible flows at low pressure, which also envisages a coupled approach^17,18^. Specifically, for heat addition to a steady, frictionless fluid flow through a constant-area duct, the theory predicts that a compressible flow can only absorb a finite amount of heat. This occurs because, under the above assumptions, the only transport mechanism available is convective transport. Hence, the fluid flow will react to heat addition by increasing the local mass flux ($\rho w$), with $\rho$ denoting the fluid density and *ω* the fluid velocity, respectively. This simultaneously leads to an increase of the convective internal energy flux (i.e. $\rho w h$ with *h* the specific enthalpy), while keeping constant the mass flow rate (steady state constraint). Consequently, heat addition will cause both supersonic and subsonic Mach numbers to approach Mach 1, resulting eventually in a thermally choked flow. Moreover, the closer the local Mach number is to the sonic value, the lower the capability of the flow to increase to local mass flux. It follows that, for subsonic flows, the amount of heat absorbed by a compressible flow is a rapidly decreasing function of the Mach number. Close to the critical point, the strong variation in the thermodynamic response functions ($\alpha _p$, $c_p$) and their mutual ratio ($\alpha _p / c_p$) may significantly alter the response of near-critical and supercritical fluids to heat addition. In order to better understand the complex interplay between fluid dynamics and thermodynamics close to the critical point, the initial model of Zierep^17^ for a pipe with constant cross-section has been extended to supercritical fluids. In a first approximation, the buoyancy force has been neglected, so that the predictions are only valid for forced convection flows, as encountered in regenerative cooling applications for rocket engines owing to the high mass flux (i.e. $\mathcal {O}(10^3)< G < \mathcal {O}(10^4)$)^4,7,13^. The paper is organized as follows. First, the results of the extended theory are presented for different fluids together with a comparison with the predictions for a perfect gas. The objectives here are twofold. The first goal is to highlight when compressible effects become relevant for SCFs. The second goal is to understand how inlet conditions and thermodynamic response functions influence the amount of heat that SCFs can absorb under the assumption of constant mass flow rate. Second, the extended model is applied to an actual HTD study for liquid rocket applications. The objective is to clarify the empirically found dependency upon $\dot{q}_w/G$ and $\alpha _p / c_p$ in the context of the coupled transport approach. Finally, a brief description of the modeling approach and validation is presented.

## Results

This section presents a comparative analysis of the response to heat addition of an ideal gas and a SCF. This allows to understand the effect of extrema in the thermodynamic response functions ($\alpha _p, \, c_p$) on the fluid capability to absorb heat and transport it by convection under steady state conditions. The following nomenclature is used. The subscripts 1 and 2 denote the conditions before and after heat addition. The non-dimensional energy ratio $Q = q/(h_1 + w _1^2/2)$ measures the amount of specific heat *q*, absorbed by the fluid, in relation to the total energy content of the flow with $w_1$ the velocity and $h_1$ the specific enthalpy prior to heat addition. As a first step, the response of gaseous and supercritical nitrogen to heat addition is investigated at two representative test conditions, respectively. The latter are reported in terms of reduced temperature $T_r = T/T_c$ and pressure $p_r = p / p_c$, where the subscript *c* denotes the critical value. Note that, once the inlet conditions are selected in terms of temperature $T_{r,1}$, pressure $p_{r,1}$ and Mach number $M_1$, also the mass flow rate $\dot{m}$ is fixed, being $\dot{m} = c_{s1} \rho _1 M_1 A$ with $c_{s}$ the adiabatic speed of sound and *A* the cross-section of the pipe. In this work, only subsonic Mach numbers are considered, because cooling channels are never operated at transonic or supersonic Mach numbers, in order to minimize dissipation and the operating pressure drop. Note, additionally, that the order of magnitude of the inlet mass flux is determined by the inlet pressure. For nitrogen at $p_{r,1} = 0.01$, this yields *G* values in the range of $1.2 \cdot 10^{-3} \le G \le 120$ (kg m$^{-2}$ s$^{-1}$), corresponding to inlet Mach numbers in the range of $10^{-5} \le M_1 \le 1$. These small values in the inlet mass flux de facto limit the local amount of heat that can be absorbed to a maximum of 0.4 (see e.g. Fig. 3a). For nitrogen at $p_{r,1} = 1.2$, *G* values in the range of $6.29 \cdot 10^{-3} \le G \le 6.29 \cdot 10^{4}$ (kg m$^{-2}$ s$^{-1}$) are obtained, which yields values for *Q* as high as 10 (see e.g. Fig. 5a). This effect could not be observed in the original model of Zierep, as the analysis extended only up to $Q=0.1$ limitedly to an ideal gas^17^. Under these assumptions, the controlling parameter for heat addition is mainly the local Mach number, which provides a measure of the flow compressibility. In this work, we extend the analysis of Zierep by providing a unified approach that includes the combined effects of the inlet conditions ($p_{r1}$, $T_{r1}$), local Mach number and ($\alpha _p / c_p$) ratio on the capacity of a fluid to absorb heat under steady state forced convection. To begin with, note that the ($\alpha _p / c_p$) ratio provides a quantitative measure of the volume variation caused by heat addition (also termed thermal dilatation), being

1 $$\begin{aligned} \frac{\alpha _p}{c_p} = \frac{1}{v} \left( \frac{\partial v}{\partial T} \right) _p \, \left( \frac{\partial T}{T \partial s} \right) _p = \frac{1}{v} \left( \frac{\partial v}{T \partial s} \right) _p \end{aligned}$$

**Fig. 2:**
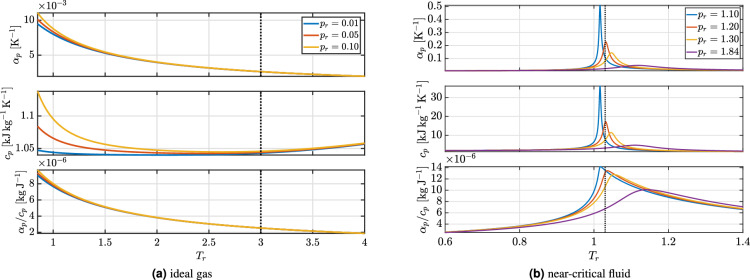
Variations of the thermodynamic response functions $\alpha _p$, $c_p$ and their mutual ratio ($\alpha _p / c_p$) for an isobaric temperature increase. Fluid: nitrogen (N$_2$). (**a**) Ideal gas behavior; (**b**) real gas behavior.

Figure 2 shows the variation of the thermodynamic response functions $\alpha _p$, $c_p$ and their mutual ratio for nitrogen behaving as an ideal gas and a near-critical dense fluid, respectively. As can be seen, in the ideal gas approximation all thermodynamic parameters are almost independent upon pressure and exhibit only a weak dependence upon temperature. Moreover, their absolute values are two orders of magnitude smaller than the corresponding near-critical values. The thermal dilatation, instead, is 2-to-7 times lower depending upon the operating temperature. These preliminary thermodynamic considerations provide a rationale for the simplifying assumptions in Zierep’s model and provide guidelines for its extension, aimed at disclosing the interplay between thermodynamics and fluid dynamics in the fluid response to heat addition.

**Fig. 3:**
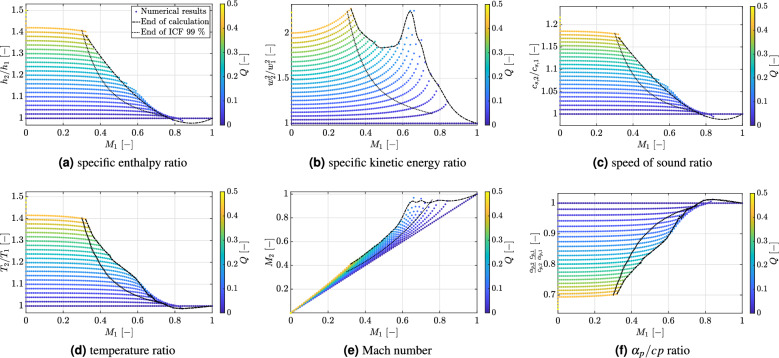
Nitrogen response to heat addition at low pressure conditions $T_{r1} = 3$, $p_{r1} = 0.01$ and $0 \le Q \le 0.5$. Mass flux range at these inlet conditions: $1.2 \cdot 10^{-3} \le G \le 120$ (kg m$^{-2}$ s$^{-1}$), corresponding to inlet Mach numbers in the range of $10^{-5} \le M_1 \le 1$. N$_2$ critical point: $T_c = 126.2$ K, $p_c = 3.39$ MPa.

At low pressure with $p_{r,1} = 0.01$ and high temperature $T_{r,1} = 3$, nitrogen can be assumed to behave as a perfect gas. Figure 3a–f visually summarize its response to heat addition. In the figures, two limiting conditions can be identified. The dashed-dotted line represents a reasonable estimation of the maximum amount $Q_{max}$ that can be added to a flow for a given Mach number $M_1$ (alias inlet mass flux at the specified operating pressure/temperature conditions). The dotted line, instead, denotes the onset of compressible effects, marked by a deviation of 1% from the incompressible solution. For *Q* values below 0.2, the incompressible flow behavior is clearly recognizable at almost all $M_1$. Its main features can be summarized as follows. The supplied energy increases the enthalpy after heat addition ($h_2/h_1 > 1$). Moreover, for constant *Q*-values, its decrease is negligible over the Mach number. This result is consistent with the fact that, for a perfect gas, the specific enthalpy is only a function of temperature. Similar considerations hold also for the speed of sound ratio ($c_{s,2}/c_{s,1} >1$). As shown in Fig. 3c, the speed of sound after heat addition is only a function of temperature and not of the inlet Mach number. In the incompressible flow region, $Q_{max}$ is constrained to roughly 0.4 irrespective of the inlet Mach number, owing to the small value of the isobaric heat capacity $c_p$ and the low inlet pressure. The fluid has therefore a diminished capacity to store energy and the low pressure conditions reduce its capacity to perform work to expand the gas. Moreover, the ratio ($\alpha _p/c_p$) decreases in state 2 upon heat addition, as shown in Fig. 3f. This means that nitrogen experiences a smaller thermal dilatation (i.e. specific volume variation) compared to state “1” for the same value of *Q* added. Under these conditions, the heat is mainly used to heat up the gas (i.e. $T_2/T_1 > 1$ in Fig. 3d). Consequently, the specific kinetic energy ratio ($w_2^2/w_1^2$) increases to compensate for the density decrease with increasing temperature, as predicted by the equation of state. This is a necessary requirement in order to fulfill the constraint of constant mass flow rate. Compressibility effects are confined in the area embedded between the $Q_{max}$ threshold and the dotted line. In this region, the trend of the ($\alpha _p/c_p$) ratio inverts. The increase in thermal dilation implies that part of the internal energy is used to expand the gas. Accordingly, the enthalpy ($h_2/h_1$) and temperature ($T_2/T_1$) ratio start to decrease with increasing $M_1$, owing to the work done by the pressure to expand the gas. This leads to a more rapid increase in the kinetic energy after heat addition. Equally, the ratio $c_{s,2}/c_{s,1}$ exhibits a slight decrease upon the onset of compressibility effects. Note, however, that the value of $Q_{max}$ in the range $0.3< M_1 < 0.6$ is not constrained by thermal choking (i.e. $M_2 < 1$), as shown in Fig. 3e. Here, the value $Q_{max}$ is determined by the local increase in thermal dilatation and the reduced capacity to perform pressure work, required to further expand the gas, owing to the low inlet pressure ($p_{r1} = 0.01$). In summary, large volume variations are not possible and this results in a decrease for $Q_{max}$ with increasing $M_1$. Please note that this effect was never reported by Zierep, who limited the analysis to $Q =0.1$. Consequently, the incompressible region appeared to have no limits in $Q_{max}$ and the compressible flow region reached immediately thermal choking upon heat addition^17^. As soon as the ratio $(\alpha _{p,2} c_{p,1}) / (\alpha _{p,1} c_{p,2})$ increases above 1, the local enhancement in thermal dilation leads to a decrease in fluid temperature upon heat addition. Recalling the definition of thermal dilatation from Eq. (1), it follows that the associated increase in specific volume can only occur at the expense of the fluid internal energy owing to the fact that the pressure does not change. For an ideal gas, this occurs only at high Mach number, where the flow is undeniably compressible. The local acceleration is such that the Mach number after heat addition is very close to one, implying that the flow is thermally choked and no further energy can be supplied. To the best of the authors’ knowledge, this is the first time that a physical explanation is provided for the well-known cooling of ideal gases upon heat addition in nearly sonic Rayleigh flows.

**Fig. 4:**
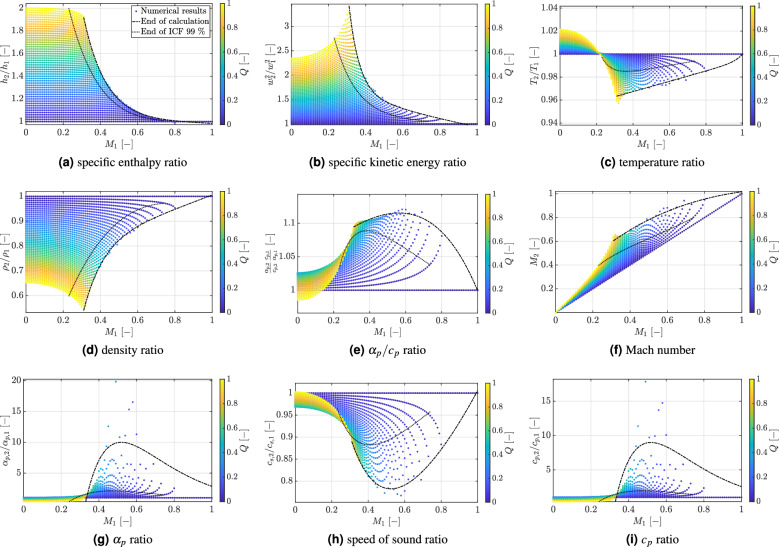
Nitrogen response to heat addition close to the critical point $T_{r1} = 1.03$, $p_{r1} = 1.2$ and $0 \le Q \le 1$. Mass flux range at these inlet conditions: $6.29 \cdot 10^{-3} \le G \le 6.29 \cdot 10^4$ (kg m$^{-2}$ s$^{-1}$), corresponding to inlet Mach numbers in the range of $10^{-5} \le M_1 \le 1$. N$_2$ critical point: $T_c = 126.2$ K, $p_c = 3.39$ MPa.

The above picture becomes more complex close to the critical point with $p_{r,1} = 1.2$ and a near-critical reduced temperature of $T_{r,1} = 1.03$ due to the increased importance of the thermal dilatation. The latter not only strongly affects the dense fluid response to heat addition, but it also imposes an additional constraints to $Q_{max}$. As it is best to proceed in a systematic way, Fig. 4a to i illustrate in detail the combined variation of flow variables and thermodynamic response functions by zooming into the range $0 \le Q \le 1$. For $M_1 \le 0.2$, the incompressible flow solution is valid for all shown *Q* values. For the specific enthalpy (Fig. 4a) and specific kinetic energy (Fig. 4b) ratios, the expected incompressible flow behavior is observed, characterized by a weak dependence upon $M_1$. The flow variables, instead, deviate significantly from the ideal gas behavior owing to the increased value of the thermodynamic response functions $c_p$ and $\alpha _p$ (see Fig. 2). Specifically, the high $c_p$ value prevents any significant increase in fluid temperature (see Fig. 4c); while the simultaneous high value of $\alpha _{p,2}$ induces large volume variations as a result of heat addition (see Fig. 4d). This increased fluid compressibility is mirrored by the steeper decrease in speed of sound, yielding $c_{s,2}/c_{s,1} \le 1$ for all *Q* values (Fig. 4h). In summary, for the incompressible flow region, enthalpy changes are primarily caused by variations of the thermal component $c_p T$ and, to a lesser extent, by pressure/density variations. This is corroborated by the behavior of the $(\alpha _{p,2} c_{p,1}) / (\alpha _{p,1} c_{p,2})$ ratio, as shown in Fig. 4e. As long as the latter is below roughly 1.025, then the energy supplied is still mainly stored in the thermal component $c_p T$ due to the negligible variation of thermal dilatation upon heat addition. Above this threshold, strong cooling effects occur (i.e. $T_2/T_1<1$). Basically, the following correlations can be observed in Fig. 4e and c: the higher $(\alpha _{p,2} c_{p,1}) / (\alpha _{p,1} c_{p,2})$ increases above 1, the stronger the resulting temperature decrease. As explained earlier, the local enhancement of thermal dilatation requires additional work to increase the fluid specific volume, which is done at the expenses of the fluid internal energy. Note that this behavior occurs even in the incompressible flow region. Hence it is directly connected to the large thermal dilatation of supercritical fluids close to the critical point. The effect is further enhanced by the compressibility of the flow at high Mach numbers, where density variations in the flow are also no longer negligible and only relatively small *Q* values can be supplied. This represents the first example of how the intrinsic compressibility of SCFs, exemplified by the larger values of the thermodynamic response functions, influences the flow response to perturbations by inducing strong counterintuitive effects, such as cooling upon heat addition.

An additional anomaly is observed in the $M_2$ response after heat addition. Again, for inlet Mach numbers in the range $0.38< M_1 < 0.6$, the limit $Q_{max}$ is attained without approaching the thermal choking condition for $M_2$, i.e. $M_2 \rightarrow 1$. This can be deduced by comparing Fig. 4a and f and represents an additional effect not included in the original Zierep’s model. This anomaly is very different compared to the case of an ideal gas at low pressure conditions, discussed above, due to the following reasons. First, for $p_{r1} = 1.2$ the inlet mass flux *G* is high enough to absorb heat up to the threshold value of $Q=10$. Second, at near-critical conditions the $(\alpha _{p,2} c_{p,1}) / (\alpha _{p,1} c_{p,2})$ ratio can achieve values significantly above 1, which is never the case for an ideal gas. To better understand the effects on the flow of this enhanced thermal dilatation, the conditions for the attainment of $Q_{max}$ are investigated in more details for three representative fluids. The overarching goal is to verify whether the behavior of nitrogen is also reproduced by other fluids close to the critical point and clarify the influence of thermal dilatation enhancement on the constraint to heat addition.

**Fig. 5:**
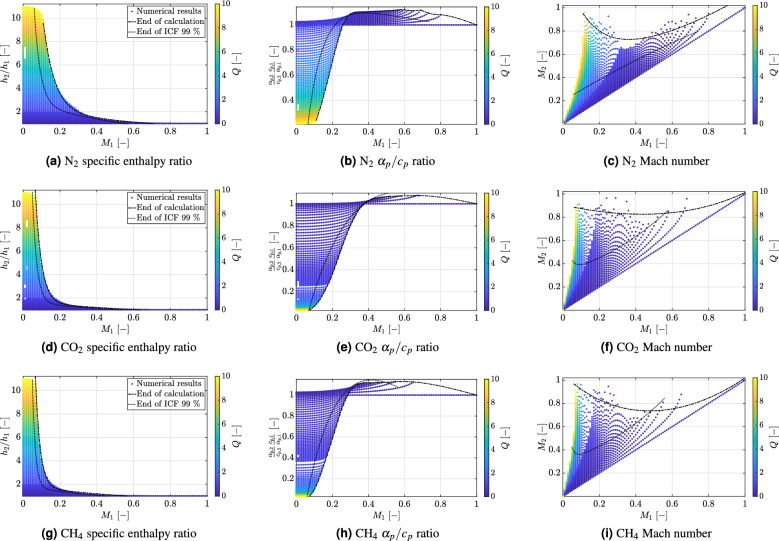
Fluid response to heat addition close to the critical point $T_{r1} = 1.03$, $p_{r1} = 1.2$ and $0 \le Q \le 10$: (**a**) to (**c**) nitrogen. Mass flux range at these inlet conditions: $6.29 \cdot 10^{-3} \le G \le 6.29 \cdot 10^4$ (kg m$^{-2}$ s$^{-1}$). N$_2$ critical point: $T_c = 126.2$ K, $p_c = 3.39$ MPa; (**d**) to (**f**) carbon dioxide. Mass flux range at these inlet conditions: $0.876 \le G \le 8.76 \cdot 10^4$ (kg m$^{-2}$ s$^{-1}$). CO$_2$ critical point: $T_c = 304.25$ K, $p_c = 7.38$ MPa; (**g**) to (**i**) methane. Mass flux range at these inlet conditions: $0.5 \le G \le 5.0 \cdot 10^4$ (kg m$^{-2}$ s$^{-1}$). CH$_4$ critical point: $T_c = 190.6$ K, $p_c = 4.60$ MPa. All mass flux values correspond to inlet Mach numbers in the range of $10^{-5} \le M_1 \le 1$.

Figure 5 shows a comparison in the response of nitrogen (N$_2$), carbon dioxide (CO$_2$) and methane (CH$_4$) at the same reduced test conditions, namely $T_{r1} = 1.03$ and $p_{r1} = 1.2$ and a significantly larger amount of energy ($0 \le Q \le 10$) added to the supercritical flow. For all fluids, this results in an earlier breakdown of the incompressible flow assumption with increasing *Q*. Note that any minor variation in Mach number in the range $0 < M_1 \le 0.2$ will cause a rapid decrease in the maximum amount of energy that can be absorbed by the supercritical fluid. This energy constraint provides a natural explanation for the occurrence of HTD and represents one of the most compelling argument for adopting a coupled approach. An additional important remark is that the decrease in $Q_{max}$ with increasing Mach number is steepest for methane and carbon dioxide compared to nitrogen. The reason is connected to the relative variation of the $\alpha _p/c_p$ ratio before and after heat addition, as shown in Fig. 5b, e and h. As can be deduced from the figures, for a given non-dimensional *Q*-value, the relative variation of the ratio $(\alpha _p/c_p)_2 \, / \, (\alpha _p /c_p)_1 = \alpha _{p,2} c_{p,1} / (\alpha _{p,1} c_{p,2} )$ is lower for nitrogen than the corresponding relative variation for methane and carbon dioxide. Consequently, upon heat addition by the amount *Q*, the volume expansion experienced by nitrogen is comparatively lower. From a practical perspective this implies that, for a given inlet state specified in terms of $M_1$, $T_{r1}$ and $p_{r1}$, nitrogen is capable to absorb significantly higher *Q* values before compressible effects become relevant. This can be deduced by comparing Fig. 5b and e, for example. Particularly interesting is the analysis of the Mach number variation after heat addition ($M_2$), shown in Fig. 5c, f and i. For $M_1 \approx 0.1$ or $M_1 > 0.7$, the $Q_{max}$ limit corresponds to the attainment of thermal choking after heat addition, i.e. $M_2 \rightarrow 1$. This is in agreement with the original model of Zierep. Specifically, the boundary condition of constant mass flow rate requires that all the heat supplied to the flow must be conducted away by convection by increasing the local mass flux. For a subsonic flow, this is achieved by expanding the flow towards $M_2 \rightarrow 1$, which provides the limiting condition for heat addition. This reasoning is valid if the condition $(\alpha _{p,2} c_{p,1}) / (\alpha _{p,1} c_{p,2} ) \le 1$ is verified. Above this threshold, the enhancement in thermal dilatation provides an additional constraint, hereafter denoted as volumetric constraint. Essentially, in a confined flow with constant mass flow rate, one cannot increase the volume indefinitely because there is not enough space to accommodate the volume variation. This limits the capability of the flow to increase the local mass flux by expanding the flow, which eventually decreases the attainable $Q_{max}$ value and represents an additional constraint to heat addition that does not occur for a perfect gas. Indeed, Fig. 5 shows the gradual transition from the thermal choking criterion for $Q_{max}$ (Zierep’s criterion) for $M_1 < 0.2$ to the volumetric constraint corresponding to a local minimum in $M_2$ when the $(\alpha _{p,2} c_{p,1}) / (\alpha _{p,1} c_{p,2} )$ ratio exhibits a steep increase above one. The intercept of the $Q_{max}$-line with the ordinate $(\alpha _{p,2} c_{p,1}) / (\alpha _{p,1} c_{p,2} ) =1$ marks the inlet Mach number $M_1^*$ above which heat addition leads to a cooling of the flow, i.e. for $M_1 > M_1^*$ it holds $T_2/T_1 < 1$. This trend is observed for all three fluids.

In summary, the above analysis provides a strong indication that the onset of HTD is not necessarily connected to the pseudo-boiling transition, as commonly suggested in literature. Instead, two concurrent factors appear to be the main controlling elements. Specifically, they are the energy constraint for heat addition to a steady compressible flow and the intrinsic high compressibility of SCFs close to the critical point, albeit not necessarily at the $c_p$-Widom line. For isobaric heat addition, the parameter measuring the enhancement in SCF compressibility is the ratio $\alpha _p /c_p$. Its macroscopic effect is twofold. First, it shifts the onset of compressible flow to Mach numbers that are normally considered incompressible at low pressure conditions. Second, the enhancement of the thermal dilation upon heat addition (i.e. $(\alpha _{p,2} c_{p,1}) / (\alpha _{p,1} c_{p,2} ) > 1$) poses an additional volumetric constraint to heat addition. Its macroscopic effect is to significantly reduce the $Q_{max}$ threshold for a given inlet Mach number, irrespectively of the attainment of thermal choking. The preceding analysis also shows that heat addition to a SCF can also result in a cooling of the fluid. Consequently, the analysis of the heat transfer efficiency cannot simply be based on the investigation of the temperature evolution, but should include a comprehensive analysis of the flow field.

## Discussion

This section discusses the capability of the coupled approach in predicting the onset of HTD in an actual engineering application. Since our model includes only forced convection, regenerative cooling in a rocket engine, operated with supercritical methane, is selected^3^. First, it is important to realize that the non-dimensional parameter *Q* is directly related to the heat flux to mass flux ratio, being

2 $$\begin{aligned} \frac{\dot{q}_w}{G}=\frac{\dot{Q}_w}{\dot{m}}= Q \, \left( \frac{w_{1}^{2}}{2} + h_{1} \right) \end{aligned}$$

with $\dot{Q}_w = \dot{q}_w A$ denoting the heat flow rate added to the fluid and $\dot{m} = G A = \rho _1 w_1 A = const$ the mass flow rate. The derivation of Eq. (2) is presented in the “Methods” section. Here, it is important to point out the implications and significance of Eq. (2). First, it shows that *Q* is not only a function of the local Mach number $M_1$, entailed implicitly in the expression for the mass flux *G*. Second, it provides a direct and natural explanation for the empirical observation that, for a given *G*, there is always a maximum value of the ratio $(\dot{q}_w/G)_{max}$ that marks the onset of HTD^4^. The latter corresponds simply to the energy constraint $Q_{max}$ that a compressible flow experiences for a given inlet mass flow rate owing to the steady state constraint that all energy absorbed by the fluid must be convected away. The intrinsic enhanced compressibility of near-critical fluids, mirrored by the large values in thermodynamic response functions, promotes the onset of compressible flow already at Mach number of the order of 0.1. This also explains why the onset of HTD correlates with the ($\alpha _p/c_p$) increase^4^, which measures the volume increase caused by heat addition. Hence, when the parameter ($\alpha _p/c_p$) increases significantly above one, compressibility effects are no longer negligible and affect the flow response in terms of both thermal and volumetric constraints. To verify the plausibility of these statements, test case S1 from Haemisch et al.^3^ is selected. Note that, for the S1 test case, HTD was experimentally detected. The test conditions are listed in Table 1. From the input parameters, it is possible to calculate the non-dimensional *Q* parameter associated to the heat flux from the wall ($Q_{w,exp}$), yielding

3 $$\begin{aligned} Q_{w,exp}= \left( \frac{\dot{q}_w A_w}{\dot{m}} \right) \, \left( \frac{1}{e_{tot,1}} \right) \end{aligned}$$

with $A_w$ the surface of the wall and $e_{tot,1} = h_1 + w_1^2 /2$. In Eq. (3), the same reference quantities as in the coupled approach were chosen to make the wall heat flux dimensionless. The dimensionless heat in the coupled one-dimensional approach can be derived from the conservation of energy, yielding

4 $$\begin{aligned} Q_{BE}= \frac{e_{tot,2}}{e_{tot,1}} -1 \end{aligned}$$

**Fig. 6:**
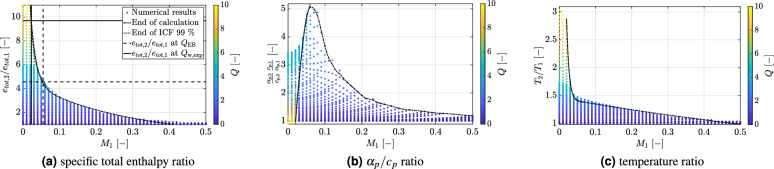
Methane response to heat addition for the test case S1^3^. Test conditions $T_{r1} = 0.73$, $p_{r1} = 1.84$ and $0 \le Q \le 10$. CH$_4$ critical point: $T_c = 190.6$ K, $p_c = 4.60$ MPa.

The respective values for $Q_{w,exp}$ and $Q_{BE}$ are listed in Table 1 with the subscript “BE” denoting the value obtained by solving the integral balance equations with the coupled approach. As can be seen, the non-dimensional heat applied to the wall is more than twice the amount of heat that can be effectively absorbed by the compressible flow. This inability to absorb all the supplied heat explains the occurrence of HTD, as observed in the experiments^3^. In order to better understand the implications of these findings, the $Q_{BE}$ and $Q_{w,exp}$ values for the S1 test case are set in relation to the regime map, obtained with the coupled model for the inlet test conditions $T_{r}$ and $p_{r}$ listed in Table 1. The results of this comparison are shown in Fig. 6. Specifically, Fig. 6a shows the specific total enthalpy ratio as function of the inlet Mach number. These non-dimensional variable can be compared directly with the non-dimensional *Q*-values calculated for the S1 test case and listed in Table 1. As can be seen, the predicted dimensionless energy absorbed by the fluid $Q_{BE}$ corresponds to the $Q_{max}$ value for a local Mach number of 0.06. The latter is intermediate between the experimental inlet and outlet Mach numbers (see Table 1). Hence, as the supercritical methane absorbs heat, it experiences a local increase in the Mach number. Simultaneously, the experimentally applied, non-dimensional wall heat flux $Q_{w,exp}$ is higher than the theoretical $Q_{max}$ values for most Mach numbers in the range $M_1 < M \le M_2$. Hence, the fluid is not capable to absorb this amount of heat and HTD occurs in agreement with the experimental observation. This result confirms the plausibility of our model predictions. Moreover, it corroborates the fact that, even in the low Mach limit (i.e. $M \le 0.1$), the amount of energy that a compressible fluid can absorb is limited by the steady-state continuity constraint. This becomes clear by observing Fig. 6b, where at $M_{1} = 0.06$ the highest $\alpha _p/c_p$ value is attained. This represents the highest possible volume variation at the given conditions to transport by convection the added energy. As discussed in the section Introduction, this finding is also consistent with empirical observation that the threshold for the onset of HTD can be formulated in terms of the product $\dot{q}_w/(G f_w) (\alpha _p /c_p)_b$. Hence, maxima in *Q* and $\alpha _p /c_p$ promote the inception of HTD. Please also note that, for the test case S1, the temperature ratio is always above 1 (see Fig. 6c), in agreement with the experimental measurements^3^.

**Table 1:** Test conditions for the HTD - S1 test case with supercritical methane. Source: Haemisch et al.^3^.

Input parameters	Inlet	Outlet
$T_r$ [-]	0.73	1.11
$p_r$ [-]	1.84	1.70
$\dot{m}$ [g s$^{-1}$]	20.1	
$\dot{q}_{w}$ [J s$^{-1}$ m$^{-2}$]	$14.5 \cdot 10^6$	

Calculated parameters	Inlet	Outlet
*wω* [m/s]	21.40	51.18
*M* [-]	0.018	0.15
$e_{tot}$ [J kg$^{-1}$]	$1.06 \cdot 10^5$	$4.84 \cdot 10^5$
$Q_{w,exp}$ [-]	8.69	
$Q_{BE}$ [-]	3.56	

CH$_4$ critical point: $T_c = 190.6$ K, $p_c = 4.60$ MPa.

**Fig. 7:**
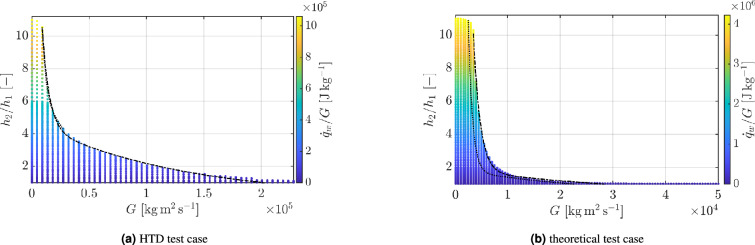
Specific enthalpy ratio for methane (a) for the test case S1^3^ - Test conditions $T_{r1} = 0.73$, $p_{r1} = 1.84$ and (b) theoretical case study. Test conditions $T_{r1} = 1.03$, $p_{r1} = 1.2$. Non-dimensional heat addition: $0 \le Q \le 10$ for both cases. CH$_4$ critical point: $T_c = 190.6$ K, $p_c = 4.60$ MPa.

To conclude this section, it is important to establish an explicit correspondence between the non-dimensional pair ($M_1$, *Q*) and the dimensional parameters (*G*, $\dot{q}/G$), typically employed in the technical applications. Figure 7 shows this comparison. First note that the values chosen for our theoretical analysis compare reasonably well with those corresponding to actual experimental cases. Second, in the S1 test case, the $(\alpha _{p,2} c_{p,1}) / (\alpha _{p,1} c_{p,2})$ ratio can reach values as high as 5, owing to the subcritical inlet temperature ($T_{r1} = 0.73$). This is because the $\alpha _p/c_p$ rapidly increase with temperature, as shown in Fig. 1b. The opposite occurs for the theoretical test case at $T_{r1} = 1.03$. As a result, $\alpha _p/c_p$ remains below one over a wide range of inlet Mach numbers (see Fig. 5b). Consequently, in the S1 test case, HTD occurs at lower $\dot{q}/G$ values owing to the additional volumetric constraint to heat addition caused by the enhancement in thermal dilation. This is also the reasons why in many experimental works the onset of HTD is reported at subcritical inlet temperatures^13^.

Despite the plausibility of the present analysis, it is important to highlight that the present conclusions are still only at phenomenological level. In reality, as the local bulk temperature and Mach number increase in the axial flow direction, the amount of heat that the flow absorbs progressively decreases with increasing Mach number. The functional dependence will be similar to the one depicted in Fig. 6a but not identical, owing to the need to take into account the local variation in flow variables, $\alpha _p$ and $c_p$ values along the axial flow direction. This is also the reason why in the experiments one observes the deterioration of heat transfer and not the abrupt attainment of an adiabatic condition. Moreover, for reliable quantitative predictions, the inclusion of viscous losses and volume expansion losses is mandatory. The latter appear not to be negligible in the case of supercritical fluids. A recent review on the topic can be found in Lamanna^2^. Despite these fundamental knowledge gaps and the still phenomenological character of the analysis, the overarching goal of this paper is to encourage further studies on the inclusion of the energy constraint to heat addition in the study of HTD in SCFs. Indeed, most studies on HTD focus on the enhancement of heat transfer from the wall to the fluid, which is strongly affected by turbulent transport and wall shear stress changes. This is definitely an important aspect, but not the only one to be considered. The present work demonstrates that low Mach number flows for SCFs clearly exhibit unique fluid dynamic behaviors. These features can only be correctly predicted and understood in the context of a compressible flow modeling approach.

## Methods

The results presented in the present work are calculated by solving the steady, one-dimensional balance equations together with the state equations for density and enthalpy. In a first approximation, only forced convection is considered, while buoyancy forces, viscous, and volume expansion losses are neglected. The model is based on the work of Zierep^17^ for a pipe with constant cross-section, which has been extended by including real gas effects through the equation of states used for density and specific enthalpy. The resulting equations are as follows:

5 $$\begin{aligned} \rho _{1} \, w_{1} - \rho _{2} \, w_{2}&= 0 \end{aligned}$$

6 $$\begin{aligned} \rho _{1} \, w_{1}^{2} + p_{1} - \rho _{2} \, w_{2}^{2} + p_{2}&= 0 \end{aligned}$$

7 $$\begin{aligned} \left( \frac{w_{1}^{2}}{2} + h_{1} \right) \, \left( 1 + Q \right) - \left( \frac{w_{2}^{2}}{2} + h_{2} \right)&= 0 \end{aligned}$$

8 $$\begin{aligned} \rho _{2} - \rho \left( T_{2} , \, p_{2} \right)&= 0 \end{aligned}$$

9 $$\begin{aligned} h_{2} - h \left( T_{2} , \, p_{2} \right)&= 0 \end{aligned}$$

Here, Eq. (5) represents the mass balance, while Eqs. (6) and (7) are the balance equations for momentum and energy, respectively. The subscripts 1 and 2 denote the conditions before and after heat addition. The non-dimensional energy ratio $Q = q/(h_{1} + w_{1}^{2}/2)$ measures the amount of specific heat *q*, absorbed by the fluid, in relation to the total energy content of the flow with $w_{1}$ the velocity and $h_{1}$ the specific enthalpy prior to heat addition. For the calculation of the specific enthalpy *h* (see Eq. (9)) and density $\rho$ (see Eq. (8)) the thermophysical property library CoolProp by Bell et al.^19^ is used, with pressure *p* and temperature *T* as input parameters.

The steady one-dimensional balance equations are solved together with the state equation through an iterative procedure using MATLAB R2023a (The MathWorks Inc.)^20^. To solve the system of nonlinear Eqs. (5–9) the MATLAB function fsolve is used applying the Levenberg-Marquardt^21,22^ algorithm. The condition before heat addition is selected as starting point. As functional and step tolerance, a value of $10^{-25}$ is implemented. After solving the system of equations, the results are checked and filtered for physical validity, namely $M_{2} \le 1$. Since in the present work only subsonic flows with a Mach number $M_{1} < 1$ are considered, the latter is a physical constraint since the maximum Mach number after heat addition is $M_{2} = 1$^17^. Note that for $M_{2} = 1$ the flow is thermally chocked^17^.

In the depicted diagrams two lines are shown together with the numerical data. The dashed-dotted line labeled ’End of calculation’ marks the last Mach number for a constant heat addition *Q* where the simulation converges and yields physically viable results ($M_{2} < 1$). The dotted line labeled ’End of ICF $99 \, \%$’ marks the onset on the compressible flow regime calculated by the following definition:

10 $$\begin{aligned} \frac{h_{2}}{h_{1}} / \frac{\frac{w_{2}^{2}}{2} + h_{2}}{\frac{w_{1}^{2}}{2} + h_{1}} > 0.99 \end{aligned}$$

The validation of the numerical approach is performed using nitrogen with the initial conditions $T_{r1} = 3$ and $p_{r1} = 0.01$ for $0< M < 1$ and $0< Q < 1$. It is important to emphasize that for these conditions a perfect gas can be assumed. The numerical results are compared with the analytical solution by Zierep^17^.

**Fig. 8:**
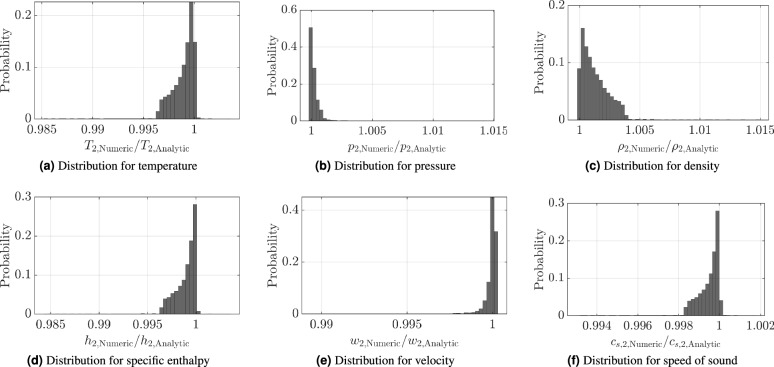
Validation of the numerical approach to heat addition compared to the analytical solution by Zierep^17^, with $T_{r1} = 3$, $p_{r1} = 0.01$. N$_2$ critical point: $T_c = 126.2$ K, $p_c = 3.39$ MPa.

The results are presented in Fig. 8 as the distribution of the ratio between numerical and analytical solutions for temperature *T*, pressure *p*, density $\rho$, specific enthalpy *h*, velocity *w*, and speed of sound $c_{s}$. The depicted distributions show that the difference between the analytical and numerical solutions is less than $1 \, \%$. For the investigated Mach number and heat addition regime. This verifies the numerical approach for subsonic flow conditions.

The derivation of Eq. (2) starting from the steady one-dimensional energy balance is shown below. Note that from Eqs. (12) to (13) the definition from Eq. (7) is applied.

11 $$\begin{aligned} \dot{m} \, \left( \frac{w_{1}^{2}}{2} + h_{1} \right) + \dot{Q}_{w}&= \dot{m} \, \left( \frac{w_{2}^{2}}{2} + h_{2} \right) \end{aligned}$$

12 $$\begin{aligned} \frac{\dot{Q}_{w}}{\dot{m}}&= \frac{w_{2}^{2}}{2} + h_{2} - \left( \frac{w_{1}^{2}}{2} + h_{1} \right) \end{aligned}$$

13 $$\begin{aligned} \frac{\dot{Q}_{w}}{\dot{m}}&= \left( \frac{w_{1}^{2}}{2} + h_{1} \right) \, Q \end{aligned}$$

## Data Availability

Correspondence should be addressed to G.L. Simulations data are available upon requests.
